# Deep Annotation of *Populus trichocarpa* microRNAs from Diverse Tissue Sets

**DOI:** 10.1371/journal.pone.0033034

**Published:** 2012-03-19

**Authors:** Joshua R. Puzey, Amir Karger, Michael Axtell, Elena M. Kramer

**Affiliations:** 1 Department of Organismic and Evolutionary Biology, Harvard University, Cambridge, Massachusetts, United States of America; 2 Research Computing, Division of Science, Faculty of Arts and Sciences, Harvard University, Cambridge, Massachusetts, United States of America; 3 Department of Biology, Pennsylvania State University, University Park, Pennsylvania, United States of America; East Carolina University, United States of America

## Abstract

*Populus trichocarpa* is an important woody model organism whose entire genome has been sequenced. This resource has facilitated the annotation of microRNAs (miRNAs), which are short non-coding RNAs with critical regulatory functions. However, despite their developmental importance, *P. trichocarpa* miRNAs have yet to be annotated from numerous important tissues. Here we significantly expand the breadth of tissue sampling and sequencing depth for miRNA annotation in *P. trichocarpa* using high-throughput smallRNA (sRNA) sequencing. miRNA annotation was performed using three individual next-generation sRNA sequencing runs from separate leaves, xylem, and mechanically treated xylem, as well as a fourth run using a pooled sample containing vegetative apices, male flowers, female flowers, female apical buds, and male apical and lateral buds. A total of 276 miRNAs were identified from these datasets, including 155 previously unannotated miRNAs, most of which are *P. trichocarpa* specific. Importantly, we identified several xylem-enriched miRNAs predicted to target genes known to be important in secondary growth, including the critical reaction wood enzyme xyloglucan endo-transglycosylase/hydrolase and vascular-related transcription factors. This study provides a thorough genome-wide annotation of miRNAs in *P. trichocarpa* through deep sRNA sequencing from diverse tissue sets. Our data significantly expands the *P. trichocarpa* miRNA repertoire, which will facilitate a broad range of research in this major model system.

## Introduction

Given the environmental and bioenergetic interest in lignocellulosic biomass, understanding the underlying molecular basis of wood formation is of great importance [1]. *P. trichocarpa*, a woody model organism with a fully sequenced genome, is uniquely positioned to address the genomics of wood formation and, as such, significant work has been done analyzing the molecular pathways leading to secondary differentiation and growth in *P. trichocarpa* [2]–[4]. In particular, Du and Groover [4] emphasized the importance of transcriptional regulation in secondary wood formation. Within this context, miRNAs have emerged as a critical regulatory component of diverse genetic programs, often by regulating transcript levels [5]. Experimental annotation of the entire miRNA component is, therefore, an essential first step to fully utilizing *P. trichocarpa* as a woody model organism and lignocellulosic feedstock.

miRNAs, a group of short (∼21 nt) non-coding RNAs in plants and animals, are known to play critical roles in diverse plant developmental processes through sequence specific gene regulation via target transcript cleavage and translational repression [6], [7]. One feature that distinguishes these molecules from other small RNAs is precise biogenesis from a stereotypical hairpin [8]. Long pri-miRNAs transcripts are trimmed by a Dicer-like 1 (DCL1) to form pre-miRNAs that fold to form stable secondary hairpin structures [6]. This pre-miRNA hairpin is further cleaved to give rise to short double stranded miRNA:miRNA* fragments [6]. The dsRNA fragment is exported to the cytoplasm where it is dissociated and the ∼21 nt miRNA is incorporated into a protein complex known as the RNA-induced silencing complex (RISC) of which ARGONAUTES are the core components [5], [6], [9]. In turn, the RISC complex, guided by the miRNA sequence, regulates specific target transcripts [5], [6].

Thorough annotation of miRNAs in an organism of interest is a critical component of transcriptome annotation. In the context of other plant systems, *P. trichocarpa* is the only woody model whose genome has been completely decoded [10]. However, despite its importance, we lack a broad and deep annotation of its miRNAs. Klevebring [11] took the first step in annotating miRNAs from *P. trichocarpa* via 454 pyrosequencing of concatenated sRNA sequences that yielded a total of 901,857 sequences. However, the sRNA library for this study was made solely from leaf tissue [11]. Two other related studies have sought to identify stress responsive miRNAs in *P. trichocarpa* [12], [13]. Sequencing of sRNAs cloned from mechanically treated xylem in Lu et al in [12] resulted in a total of 898 sequences while sequencing of cloned sRNAs from abiotically treated stress samples and mechanically stressed samples resulted in a total of 2648 and 1179 unique sequences, respectively [13]. While both of these studies made significant advances in our understanding of miRNAs, progress in next-generation sequencing allows us to study sRNAs at significantly greater depth.

The goal of this study is to expand the miRNA annotation depth of *P. trichocarpa* by sampling across a diverse set of tissue types, including the first sampling of reproductive tissue, and using deeper sequencing approaches. We have also analyzed publicly available previously unannotated *P. trichocarpa* sRNA sequencing runs from xylem, mechanically treated xylem, and leaves. By examining these datasets in concert, we gain a better understanding of the complexity of *P. trichocarpa* miRNA expression profiles and enable the identification of xylem-enhanced miRNA-target interactions.

## Results

### sRNA Sequencing Statistics Overview

Four individual sRNA libraries were analyzed. A pooled sRNA library prepared from growing vegetative apices, male flowers, female flowers, female apical buds, and male apical and lateral buds was sequenced using the SOLiD ABI platform. Leaf-specific, xylem-specific, and mechanically treated xylem (MTX)-specific sRNA libraries available for download from http://smallrna.udel.edu/ and Gene Expression Omnibus (GEO) were also analyzed. Table 1 summarizes the read counts obtained from these four sequencing runs. MTX mechanical treatment and sample collection was performed as described in Lu [13].

**Table 1:** Summary statistics of smallRNA sequencing.

	Pooled	Pooled	Xylem	Xylem	MTX	MTX	Leaves	Leaves
	Total	Unique	Total	Unique	Total	Unique	Total	Unique
Initial	5,529,597	3,551,828	4,558,622	2,122,939	3,972,826	1,039,594	4,472,811	1,410,957
tRNA/rRNA filtered	4,260,646	3,284,769	3,880,670	2,102,289	2,552,858	1,015,249	3,566,778	1,381,588
Genome filtered	1,032,498	499,064	2,592,554	1,138,249	1,581,475	397,633	2,494,937	708,989

**Figure 1:**
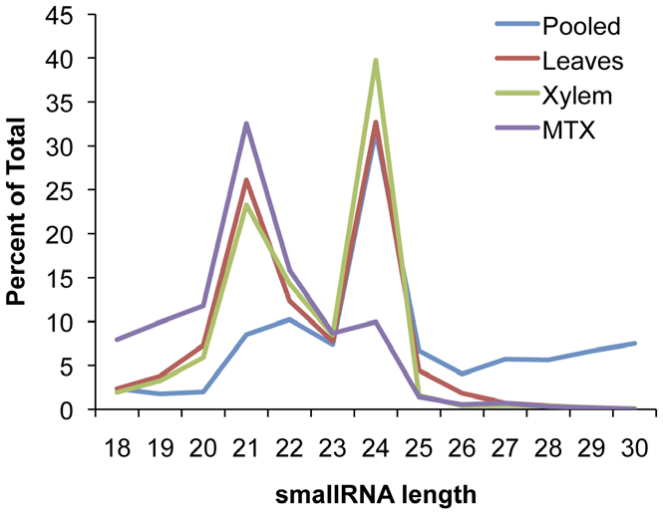
Length distribution of smallRNA reads. Length distribution of smallRNAs of obtained from four individual sequencing runs after adaptor removal but prior to filtering.

The data was analyzed using the UEA sRNA toolkit [14] in conjunction with the most recent *P. trichocarpa* genome assembly (v.156, available from http://www.phytozome.net/) as a reference [10]. Adaptors were removed from SOLiD sequences and color-space sequence was converted to base-space using custom perl scripts (the three downloaded Illumina libraries already had their adaptor sequences removed). Filtering to remove tRNA and rRNA was performed. These reads were then mapped against the reference *P. trichocarpa* genome and only perfect matches were allowed (Table 1). Length distributions before filtering are shown in Figure 1.

### microRNA Annotation

Filtered sRNA datasets were uploaded individually into the miRCAT pipeline [14]. The mirCAT pipeline annotates miRNA based on expressed sRNA sequences and stable hairpin structures [14]. miRNA annotation was performed according to criteria described in [8]. A total of 276 miRNAs were identified from these four datasets (Tables S1, S2, S3, S4). The sequence and genomic location of all known *P. trichocarpa* miRNAs were downloaded from miRBASE version 17 [10], [15]–[17] and these previously annotated miRNAs were overlaid on the new dataset. This allowed us to compare the genomic locations extracted from the gff annotation file (available for download from miRBASE) to the sequences of miRNAs annotated in the current study [15]–[17]. Based on this, we have identified a total of 155 new miRNAs. There are a total of 234 P. trichocarpa miRNAs currently deposited on miRBASE (v18), however, only 198 of these miRNAs have genome coordinates. Of the 198 miRNAs *P. trichocarpa* already annotated in miRBASE, this study identifies 122 and misses 76.

**Figure 2:**
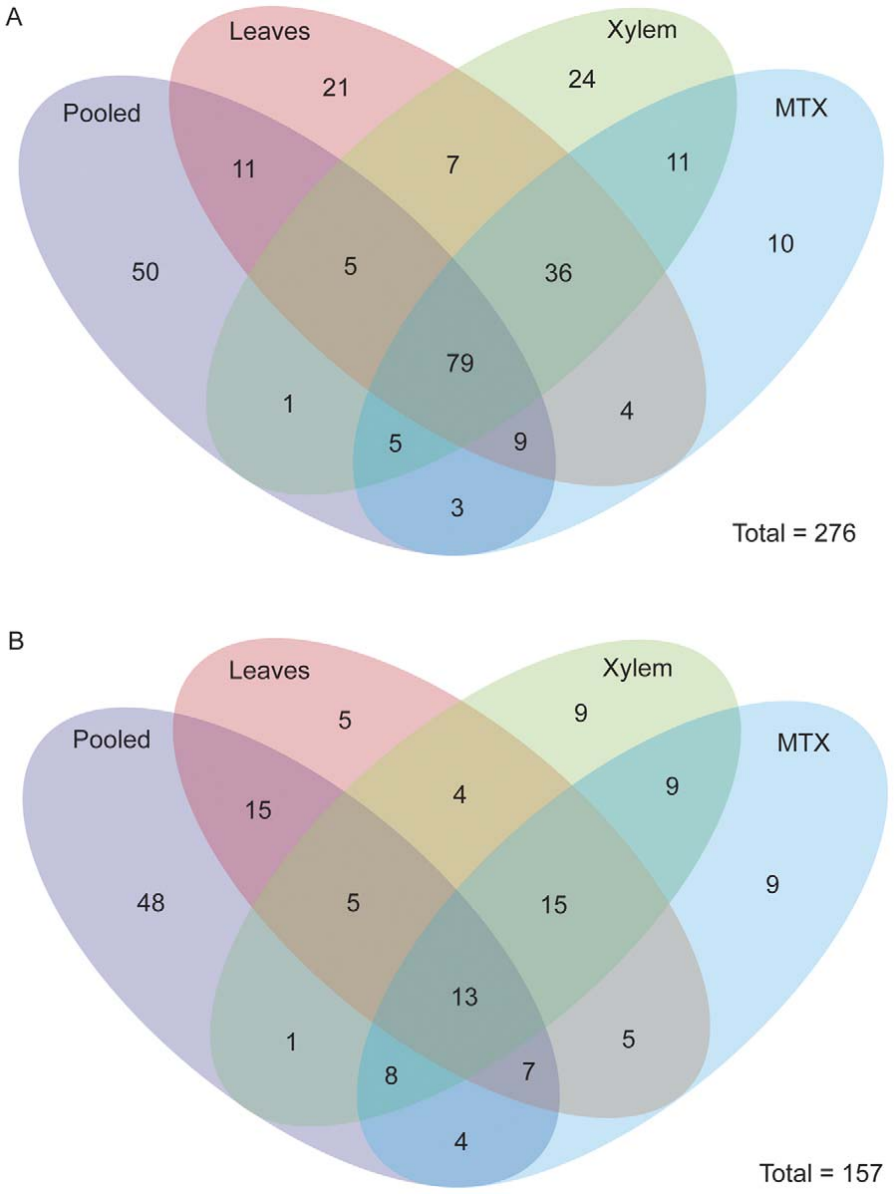
Venn diagram of miRNAs identified in four smallRNA sequencing runs. (A) Venn diagram of all miRNAs identified in a pooled, leaves, xylem, and mechanically treated xylem (MTX) samples. Over 25% of miRNAs (N = 85) were identified in all four samples. (B) To add a second level of filtering, only miRNAs with a sequence miRNA* sequence were overlaid in a venn diagram. It is interesting to note miRNAs present in all four samples dropped to 8% when the miRNA* filtering criteria was was applied. More miRNAs with miRNA* appear to be enhanced in tissue specific contexts.

In order to understand the similarity and differences between the new sRNA sequencing runs, we created a venn diagram comparing presence-absence of miRNAs across the four datasets (Figure 2A). We annotated 164 miRNAs from the pooled dataset, 173 from leaves, 169 from xylem, and 158 from mechanically treated xylem. miRNAs specific to *P. trichocarpa* were identified by looking for a matching miRNA (within 4 mismatches) anywhere in green plants in miRBASE. It is important to note that in this context, *P. trichocarpa* specificity is based on failure to annotate a specific miRNA from other genomes, which does not necessarily imply absence from other plant genomes. A total of 110 *P. trichocarpa* specific miRNAs were identified. These include 36, 53, 51, and 37 *P. trichocarpa* specific miRNAs identified from pooled, leaf, xylem, and mechanically treated xylem, respectively.

To add another level of stringency, we then asked which of these miRNAs has a corresponding miRNA* sequence (Figure 2B). A total of 157 miRNA sequences with a corresponding miRNA* were identified, including a total of 33 *P. trichocarpa* specific miRNAs. The majority of miRNAs with a miRNA* (124 of 157) could be grouped into more broadly conserved miRNA family (as defined by a miRNA family presence in other green plant species on miRBASE). Perhaps not surprisingly, the pooled tissue sample showed the highest percentage of miRNA annotated with a corrresponding miRNA* sequence - 62% (102/164). The leaf, xylem, and MTX annotated miRNAs only showed a corresponding miRNA* sequence 39% (69/173), 37% (64/169), and 44% (70/158) of the time, respectively.

### miRNA-target prediction

miRNA target prediction was performed using the psRNAtarget predictor [18]. For target prediction, the most recent *P. trichocarpa* coding sequences were downloaded from Phytozome. Predicted miRNA-target interactions are reported with the expectation score as originally defined by [19]. Expectation scores are dependent on degree of miRNA-target complementarity. Perfect complementary miRNA-target binding sites receive an expectation score of 0 while mismatches or G-U base-pair wobbles in the miRNA-target site increase the expectation score. Target predictions for miRNAs based on each sRNA library (pooled, leaf, xylem, and MTX) are available in the supplement (Tables S5, S6, S7, S8).

### Expression of miRNAs

**Figure 3:**
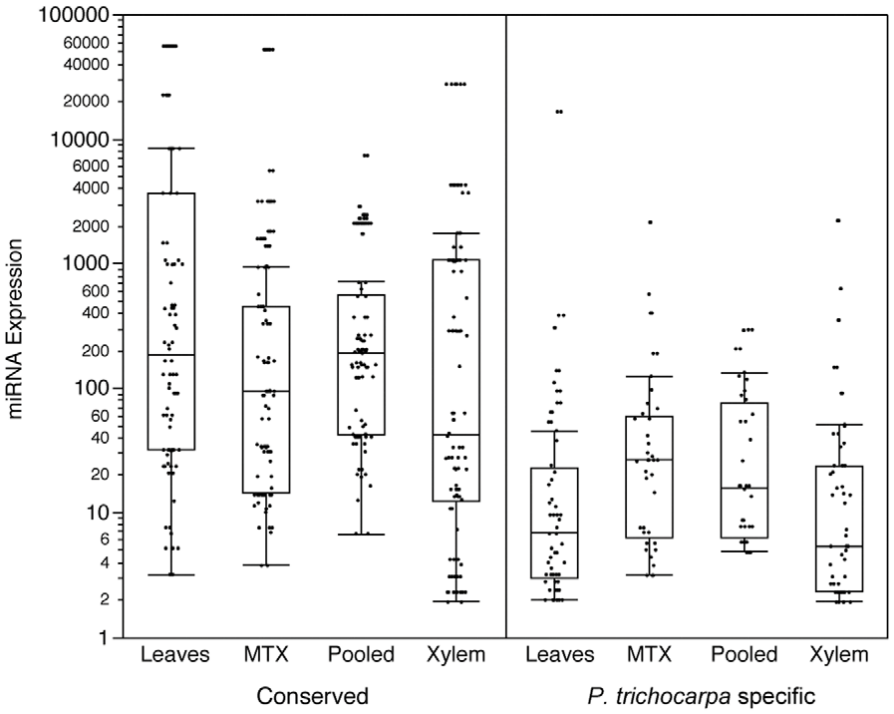
Expression of miRNAs. miRNA expression is shown in normalized units according to [20]. [miRNA expression = (Raw Abundance)/(Number of Mappable Reads)×1,000,000]. Conserved: miRNAs identified outside of *P. trichocarpa* in at least one other green plant species on miRBASE. *P. trichocarpa* specific: miRNAs only identified in *P. trichocarpa*.

In addition to miRNA annotation, miRNA expression data can be recovered via high-throughput sRNA sequencing datasets. The raw abundance of every miRNA annotated for each dataset was determined. To account for variations in sequencing depths between libraries, raw abundance was divided by the total number of perfectly mappable reads and multiplied by a constant: [miRNA expression = (Raw Abundance)/(Number of Mappable Reads)×1,000,000] [20]. As has been observed in other plant species [21], [22], more broadly evolutionarily conserved miRNAs are expressed at higher levels that *P. trichocarpa* specific miRNAs (Figure 3).

**Figure 4:**
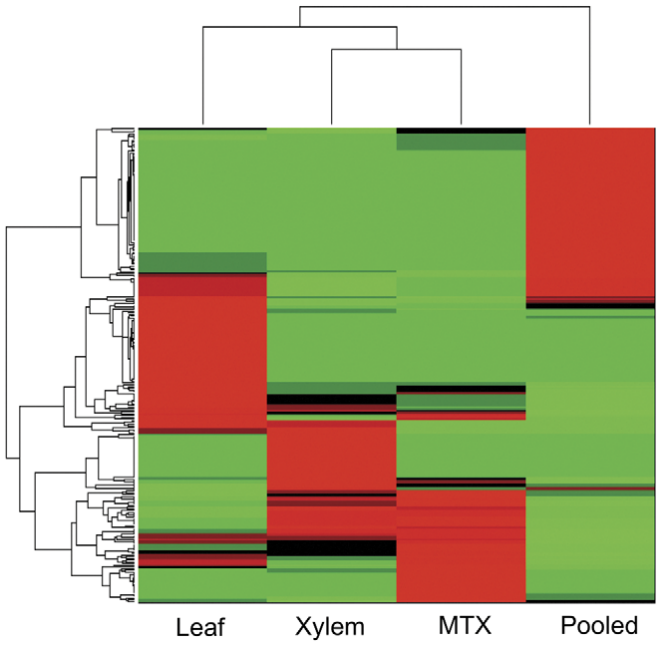
miRNA expression clustergram. Clustering of smallRNA sequencing runs by miRNA expression. [miRNA expression = (Raw Abundance)/(Number of Mappable Reads)×1,000,000] reveals that xylem and mechanically treated xylem (MTX) are most similar. MATLAB *clustergram* algorithm was used for expression clustering.

Hierarchical clustering of the four sequenced datasets based on annotated miRNAs and expression levels of these miRNAs was performed using MATLAB's *Clustergram* algorithm (Figure 4) [23], [24]. Hierarchical clustering indicates that the xylem and MTX are the most similar datasets based on miRNA expression patterns (Figure 4).

### Xylem and MTX miRNAs and their targets

All plant cell walls are composed of essentially the same basic components [2]. What commonly distinguishes one cell wall, and often one cell type, from another is the proportion and arrangement in which these building blocks are deposited [2]. Understanding the genetic regulation that controls the deposition process provides insight into the regulation of secondary and primary wall biosynthesis and holds the potential to facilitate manipulation of the process, a subject with important economic potential. For these reasons, we would like to highlight several predicted miRNA-target interactions that are uniquely associated with xylem and MTX. A total of 57 xylem-enriched miRNAs were identified in this study, of which 11 miRNAs were enriched in MTX. Of the xylem- or MTX-enriched miRNAs, 12 can be grouped into more broadly conserved miRNA families. One MTX-enriched miRNA is more broadly conserved while six more broadly conserved miRNAs are enriched in xylem. Below we discuss the potential significance of predicted miRNA-targeted genes that have been implicated in wood formation. All predicted miRNA-target interactions described below are for miRNAs that to-date have only been identified in *P. trichocarpa*.

#### ptc-miRX50 targeting of XTH16

A MTX-specific miRNA, ptc-miRX50, is predicted to target XTH16, which encodes a xyloglucan endotransglycosylase/hydrolase (XTH). This predicted interaction has a category score of 3.5 and ptc-miRX50 is expressed at a moderate to high level (normalized expression of 30.4 – Figure 3). This predicted interaction in MTX is of particular interest given the role of xyloglucan in tension wood. Tension wood is a special type of wood that contains gelatinous fibers (G-fibers), which are found on the upper side of branches and contain an increased-proportion of highly-oriented cellulose. As the cellulose fiber cells swell, a hoop-stress is generated resulting in the contraction of the entire G-fiber. The asymmetric distribution of G-fiber cells on the tree limb as a whole - the G-fiber cells being concetrated on the top of the branch - results in a drooping stem being pulled up when the G-fibers contract longitudinally. In addition to highly oriented cellulose, xyloglucan has been found to be the predominant component of the cellular matrix of G-fibers [25] and both *in situ* hybridization and antibody labeling has localized XTH to the G-fiber cells [25]. The biochemical function,and localized expression of XTH led Nishikubo [25] to hypothesize that XTH could play a role in repairing xyloglucan cross-links as the G-fibers are shrinking in tension wood. It is possible, therefore, that ptc-miRX50 plays a role in G-fiber formation and function by modulating levels of XTH16.

#### Predicted targeting of NAC domain transcription factors

In addition to predicted targeting of XTH16, ptc-miRX50 (MTX-enriched), is predicted to target a NAC domain transcription factor, *NAC083*, with an expectation value of 3.5. A second NAC domain transcription factor, *NAC050*, is also a predicted target of the xylem-specific miRNA, ptc-miRX87 (normalized expression level 4.6 and target expectation score of 3.5 – Figure 3). While NAC transcription factors are known to play essential roles in regulating secondary growth [26], [27], little is known about *NAC050* or *NAC083* in *P. trichocarpa*. It is interesting to note that while certain NAC genes are known targets of the miR164 family [6], neither *NAC050* nor *NAC083* are targets of this deeply conserved miRNA family. Furthermore, the predicted miRNA binding sites of ptc-miRX87 and ptc-miRX50 to *NAC050* and *NAC083*, respectively, are not conserved across other related NAC genes in the angiosperms.

A third miRNA, xylem-enriched ptc-miRX73, is predicted to target a vascular related NAC transcription factor called *VND7* with a target expectation score of 4.5. ptc-miRX73 has a normalized expression value of 3.1 (Figure 3). *VND7*, a NAC domain transcription factor involved in xylem vessel differentiation [28], has been a gene of considerable interest in wood formation [26], [27]. A recent paper by Yamaguchi [28] identified a range of genes that are directly regulated by VND7, including several IRX genes and an *XCP1* cysteine protease. *IRX* genes play a role in secondary wall formation [29] while *XCP1* genes play a role in programmed cell death [30].

#### ptc-miRX41 targeting of a cellulose synthase CSLD4

Cellulose is a basic component of plant cell walls that is produced by the cellulose synthase complex. ptc-miRX41 (MTX enriched) is predicted to target a cellulose synthase gene, CSLD4, with an interaction score of 3.5. ptc-miRX41 is specifically expressed at a moderate level in MTX (normalized expression of 6.9 – Figure 3).

## Discussion

Through the use of high-throughput sRNA sequencing, we have significantly expanded the breadth and depth of miRNA annotation in *P. trichocarpa*, annotating a total of 155 new miRNAs that are now deposited in miRBASE. This includes the first sampling of reproductive tissue in *P. trichocarpa*. The dramatic improvement in the breadth of tissue examined greatly increases the significance of identified xylem- and MTX-enriched miRNA-target interactions. These are likely to be of considerable economic and bioenergentic interest given their potential role in regulating wood development. Thus, the public availability of these datasets will promote a wide range of research in a critical model for woody plants.

## Materials and Methods

### Pooled sRNA library sequencing

sRNA sequencing of pooled tissue was performed using SOLiD ABI sequencing technology. The pooled tissue included growing vegetative apices, male flowers, female flowers, female apical buds, and male apical and lateral buds was sequenced using SOLiD ABI platform from *P. trichocarpa*. The diversity of the tissue used to make the pooled library required a variety of growth conditions including field, greenhouse, and regulated growth chambers. RNA was collected used the Plant RNA Purification Reagent (Invitrogen). The library was prepared with the SOLiD Total RNA-Seq kit using the small RNA variant protocol per the manufacturer's instructions. Adaptor sequences were removed and color-space converted to base-space with custom perl scripts.

### Tissue specific sRNA libraries

Tissue specific sRNA sequencing was downloaded from GEO: leaf specific sRNA, GSM717875; xylem sRNA, GSM717876; and mechanically treated xylem, GSM717877 [31]. Plant RNA Purification Reagent (Invitrogen) was used to collect RNA and libraries were prepared by Illumina (Hayward, CA). Illumina SBS sequencing technology was used to sequence these libraries. Adaptor trimmed sequences were downloaded and used for analyses.

### miRNA annotation

The most current *P. trichocarpa* genome, version 156, availble on www.phytozome.com, was used as a reference. Annotation of miRNAs was performed using mirCAT [14]. miRCAT is an online tool developed to annotate miRNAs based on next-generation high-througput sequencing data. Default miRCAT [14] options were used for initial miRNA annotation. Default miRCAT parameters are as follows: (1) tRNA and rRNA matches removed; (2) Minimum raw abundance, 5; (3) Minimum sRNA size, 18; (4) Maximum sRNA size, 24; (5) Maximum number of genomic hits, 16; (6) Minimum window size for hairpin folding test, 150; (7) Maximum number of non-overlapping hits at a given locus, 3; (8) Maximum percentage of unpaired bases in a folded hairpin, 50; (9) Maximum total length of overlapping sRNAs, 70; (10) Percentage of sRNAs that must be in the same orientation at a given locus, 90; (11) Minimum number of paired bases in the miRNA region of a folded hairpin, 17; (12) Maximum consecutive unpaired bases in miRNA region, 3; (13) Minimum G/C percentage in miRNA, 10; and (14) Minimum possible length of hairpin, 75.

These requirements adhere to the criteria of plant miRNA annotation described by Meyers et al. [8] except for the minimum number of paired bases (17 nt) in the miRNA region of a folded hairpin and miRNA processing precision. Meyers et al. [8] requires that the there be no more than 4 unpaired bases in the miRNA region of the hairpin. For 21 nt miRNA the miRCAT pipeline adheres to Meyers et al. [8] criteria. However, given the requirement of a minimum of 17 paired bases, it is possible that a 22 nt miRNA has 5 unpaired bases in the miRNA region. To account for this possibility and verify that all annotated miRNAs adhere to Meyers et al. [8] folding of all pre-miRNAs was performed and unpaired bases of the miRNA region counted.

miRNA processing precision is a key criteria in annotating miRNAs [8] and is not explicitly taken into account in the miRCAT pipeline. Processing precision was calculated using custom perl scripts. smallRNA reads from a given library were aligned to miRNA precursor sequences (Tables S1, S2, S3, S4). Raw abundance values of each read were summed for half of the hairpin. The raw abundance value of the predicted miRNA was divided by the total raw abundance for half of the hairpin to give a processing precision value. All miRNAs with processing precision below 25% were thrown out. This criteria guarantees that the miRNA sequence represents a quarter or greater of the total reads on miRNA half of the hairpin.

### Target prediction

Target prediction was performed using psRNAtarget predictor available at http://biocomp5.noble.org/psRNATarget/ [18]. Targets were predicted for all miRNAs identified and are available in (Tables S5, S6, S7, S8).

## Supporting Information

Table S1 **miRNAs annotated from Pooled sRNA library.**
(XLSX)

Table S2 **miRNAs annotated from Leaf sRNA library.**
(XLSX)

Table S3 **miRNAs annotated from Xylem sRNA library.**
(XLSX)

Table S4 **miRNAs annotated from MTX sRNA library.**
(XLSX)

Table S5 **Targets predicted for miRNAs identified in Pooled library.**
(XLSX)

Table S6 **Targets predicted for miRNAs identified in Leaf library.**
(XLSX)

Table S7 **Targets predicted for miRNAs identified in Xylem library.**
(XLSX)

Table S8 **Targets predicted for miRNAs identified in MTX library.**
(XLSX)
